# Hormonal Therapy for Infantile Spasms: A Systematic Review and Meta-Analysis

**DOI:** 10.3389/fneur.2022.772333

**Published:** 2022-02-10

**Authors:** Shiqi Guang, Leilei Mao, Linxiu Zhong, Fangyun Liu, Zou Pan, Fei Yin, Jing Peng

**Affiliations:** Department of Pediatrics, Xiangya Hospital, Central South University, Changsha, China

**Keywords:** infantile spasms, west syndrome, hormonal therapy, ACTH, corticosteroids

## Abstract

**Objective:**

The limitations of adrenocorticotrophic hormone (ACTH) treatment for infantile spasms (ISs), such as high costs, limited availability, and adverse effects (AEs), make it necessary to explore whether corticosteroids are optimal alternatives. Many other compelling treatments have gone through trials due to the suboptimal effectiveness of hormonal therapy. A systematic review and meta-analysis were performed to evaluate the effectiveness and safety of hormonal therapy for patients with ISs.

**Methods:**

EMBASE, Ovid MEDLINE, Cochrane Central Register of Controlled Trials (CENTRAL), and online registers were searched through April 2021 for randomized controlled trials (RCTs).

**Results:**

A total of 19 RCTs (*N* = 1,279) were included. There was no significant difference in the effectiveness of oral corticosteroids and ACTH in electro-clinical response (risk ratio [RR] = 0.85, 95% CI 0.41–1.76). Low-dose ACTH had similar effectiveness in electro-clinical response compared to usual-dose group (RR = 0.94, 95% CI 0.60–1.47) but conferred a lower risk of AEs (RR = 1.71, 95% CI 1.08–2.71). ACTH was more beneficial in controlling spasms than vigabatrin (VGB) (RR = 1.31, 95% CI 1.05–1.64) for patients without tuberous sclerosis complex (TSC). All RCTs were connected through network meta-analysis, and we found that ketogenic diet (KD), zonisamide, methylprednisolone, or combined treatment of hormonal therapy with topiramate (TPM) or pyridoxine was not different in electro-clinical response compared to usual-dose ACTH.

**Conclusion:**

Our analysis showed that oral corticosteroids could be optional alternatives when ACTH is not applicable, and ACTH is more beneficial for patients without TSC. Moreover, low-dose ACTH is recommended due to comparative effectiveness but lower risk of AEs. However, due to the high heterogeneity of included patients and treatment protocols, these results must be interpreted with caution. RCTs with multicentric involvement and larger sample size are needed for solid evaluation of other alternative treatments.

## Introduction

Infantile spasms (ISs), also known as West Syndrome, with a minimal incidence of 0.25 per 1,000 live births (1), are early-onset developmental and epileptic encephalopathy. It is characterized by a triad of epileptic spasms, neurodevelopmental regression, and hypsarrhythmia on the electroencephalogram (EEG), mainly occurring at the age of 3–12 months, with a peak occurrence around 4–6 months (2). After developmental arrest or regression, about 70% of infants become severely disabled both physically and intellectually and can develop multiple types of refractory seizures in the future. Currently, hormonal therapy [adrenocorticotrophic hormone (ACTH) and corticosteroids] and vigabatrin (VGB) are the most effective treatments for ISs. ACTH is the preferred first-line treatment for most patients with ISs, but ACTH cost is continuing to rise, and it is difficult to obtain in some countries (3, 4). Moreover, the incidence of adverse effects (AEs) of ACTH treatments is high, and thus, corticosteroids are substitutes that are well worth being explored for the treatment effectiveness, especially for low- and middle-income countries. However, hormonal therapy is partially effective. The responder rate of hormonal therapy varied from 37 to 87%, and the relapse rate can be up to 66% (5–12). Therefore, other treatments, including antiepileptic drugs (AEDs) and the ketogenic diet (KD), are also actively investigated. To date, optimal treatments of ISs are still open for investigation and discussion. Thus, we performed this systematic review and meta-analysis to provide more evidence for clinical decisions in choosing other treatments for ISs over ACTH.

## Methods

### Inclusion Criteria of Studies for Our Analysis

#### Participants of Studies

Patients who were newly diagnosed with ISs without receiving any hormonal treatments before enrollment were eligible for our review and analysis.

#### Intervention and Trial Design of Studies

Based on our knowledge about hormonal therapy for ISs, the topics of relevant clinical trials could be grouped into categories as follows: (1) ACTH vs. oral or intravenous corticosteroids, (2) comparison of different dosages of hormonal therapy, (3) hormonal therapy vs. VGB, (4) hormonal therapy vs. other treatments, and (5) hormonal therapy alone vs. combined treatments. Randomized controlled trials (RCTs) with double-blind, single-blind, and open-label designs that studied the above topics were considered for inclusion.

#### Outcomes of Studies

Studies included in our analysis must report the complete cessation of spasms as one of the clinical end points. Remission of hypsarrhythmia on EEG, relapses, as well as developmental outcomes were expected to report, but these outcomes were dispensable for a study to be included or not.

#### Safety and Tolerance

The AEs were documented.

### Data Sources

This study was conducted and reported according to the Preferred Reporting Items for Systematic Reviews and Meta-Analyses (PRISMA) guidelines (13). The electronic databases, including EMBASE, Ovid MEDLINE, and Cochrane Central Register of Controlled Trials (CENTRAL), were searched from database inception to April 26, 2021, to identify the published data. The search strategies (shown in Supplementary Material 1) were developed specially for each database, and the keywords were adapted according to the configuration of each database. Online trial registers, including NIH ClinicalTrials.gov, metaRegister of Controlled Trials, and WHO International Clinical Trials Registry Platform (ICTRP), were searched to identify ongoing trials or unpublished trials. The search terms, including “infantile spasms” and “West Syndrome,” were used when searching online registers. Searches were not limited by language, date, or publication status.

### Assessment of Risk of Bias, Data Extraction, and Synthesis

The risk of bias of RCTs was assessed by two independent authors using the Cochrane Collaboration's tool (14). Briefly, six domains of bias, namely, selection bias, performance bias, detection bias, attrition bias, reporting bias, and other bias, were rated. Studies with all domains to be rated as low risk were considered to have a low risk of bias. If one or more domains were rated as unclear risk, and the other domains were at low risk of bias, the studies were considered as having unclear risk of bias. One study was considered to have a high risk of bias if one or more domains were rated as having a high risk of bias.

The data, including authors, publication year, sex ratio, treatment protocols, the number of patients on each treatment arm, the number of patients achieving spasm cessation and electro-clinical response, as well as AEs, were extracted by one author and confirmed by two other review authors. The electro-clinical response was defined as the complete cessation of spasms with EEG remission of hypsarrhythmia. Review Manager version 5.3 was used for calculations of data synthesis. For dichotomous outcomes, such as electro-clinical response and AEs, the risk ratio (RR) with 95% CI was calculated after data synthesis. Statistical significance for RR was defined as 95% CI not containing 1. For crossover RCTs, only results of the initial stage were synthesized and analyzed.

### Network Meta-Analysis

To gain a better understanding of the performance of treatments with no head-to-head trials with ACTH, a network was generated through joint treatments. Network meta-analysis was conducted using MetaXL (version 5.3). Each direct comparison was wrapped in the MAInputTable function (IOType = “NumRR” and Method = “IVhet”), and then the MANetwork function was used to perform network meta-analysis. Consistency H was calculated at the same time.

## Results

### Study Selection and Characteristics

**Figure 1:**
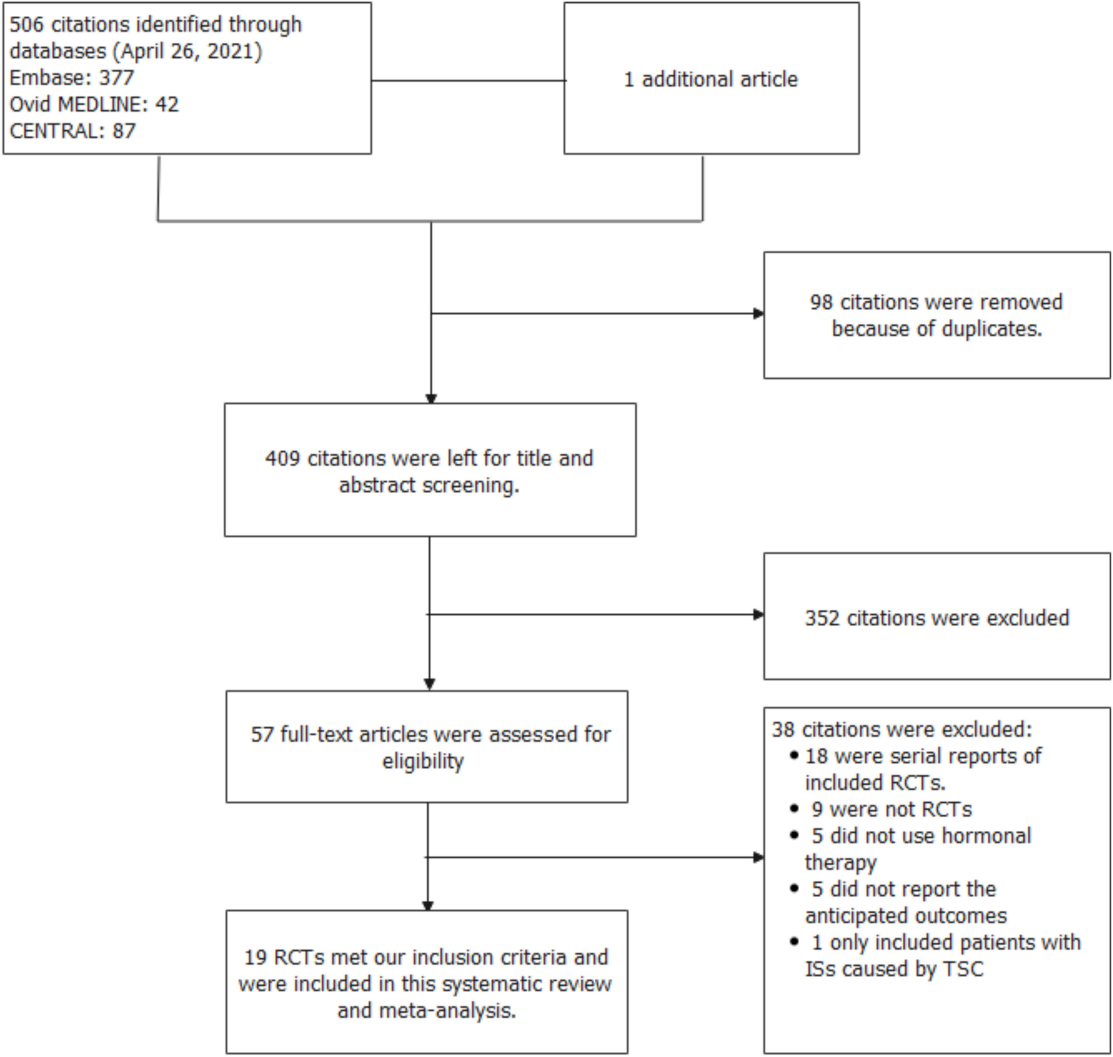
Flow diagram of the study selection process.

**Figure 2:**
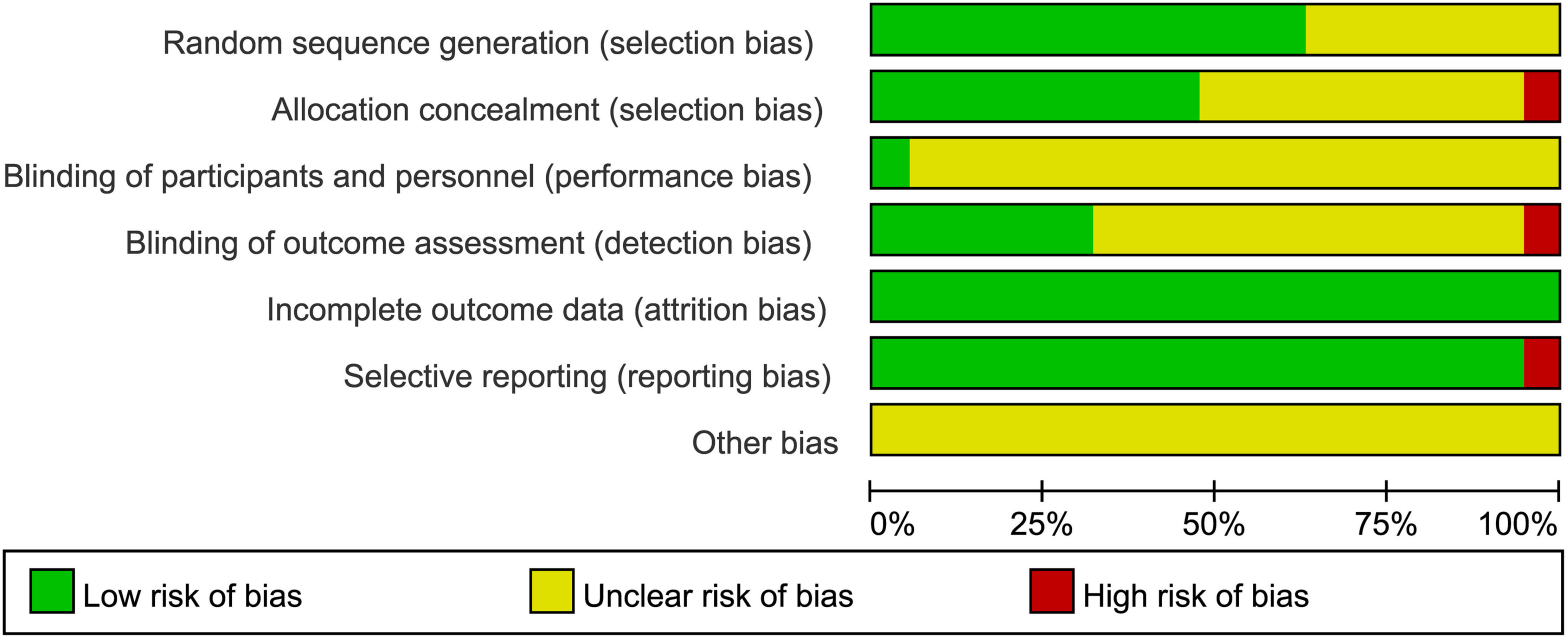
Judgments about each risk of bias item presented as percentages across all included studies.

In total, 507 records were identified in the initial search; 98 were removed due to duplications. Through title and/or abstract screening, 352 were excluded. After perusing full texts, 38 records were excluded, and the reasons for exclusion are listed in Figure 1. Overall, 19 RCTs (comprising 1,279 patients), which were conducted between 1983 and 2021, met our inclusion criteria. The characteristics of the included studies are presented in Table 1. All studies were alleged as RCTs; however, only twelve and nine studies provided sufficient descriptions on random sequence generation and allocation concealment, respectively. We accepted that blinding of participants and care providers could not be done in most circumstances as two interventions were given through different routes. Only one study, Hrachovy et al. (5), used a double-blind design, and five studies applied a single-blind design by masking personnel to treatment protocols when analyzing EEG results. The remaining studies were open-label randomized trials. Judgments about each risk of bias item are presented in Figure 2. Although only nine out of 19 studies reported electro-clinical response (defined as complete cessation of spasms with EEG remission of hypsarrhythmia) as the primary outcome, corresponding EEG results were documented as one of the secondary outcomes in all other studies except for Omar et al. (19), making meta-analysis of electro-clinical response possible. Time intervals between treatment initiation and electro-clinical response varied, but 2 weeks after treatment initiation was the most common time point to evaluate treatment effectiveness.

**Table 1:** The characteristics included randomized controlled trials (RCTs).

**Author, year**	**Trial design**	**No. of patients**	**Male/female ratio**	**Intervention 1 (No. of patients)**	**Intervention 2 (No. of patients)**	**Primary outcome**	**Secondary outcome**	**Time interval between primary outcome and initiation of treatment**
**ACTH VS. LOW-DOSE CORTICOSTEROIDS**								
Hrachovy et al. 1983 (5)	Double blind	24	NR	ACTH gel 20 U/day and prednisone placebo for 2 weeks (12)	Oral prednisone 2 mg/kg/day and ACTH gel placebo for 2 weeks (12)	Spasm cessation and EEG remission	AEs	2–6 weeks
Baram et al. 1996 (7)	Single blind	29	0.7	ACTH gel 150 U/m^2^/day for 2 weeks (15)	Oral prednisone 2 mg/kg/day for 2 weeks (14)	Spasm cessation and EEG remission	NR	2 weeks
Sardar et al. 2019 (15)	Open label	70	3.4	ACTH 150 IU/m^2^/day for 2 weeks (35)	Oral prednisolone 2 mg/kg/day for 4 weeks (35)	Spasm cessation and EEG remission	NR	2–8 weeks
**ACTH VS. HIGH-DOSE CORTICOSTEROIDS**								
Lux et al. 2004 (9)	Open label	55	1.4	Synthetic ACTH depot 40–60 IU on alternative days for 2 weeks (25)	Prednisolone 40–60 mg/kg/day for 2 weeks (30)	Spasm cessation	Time taken to response; EEG remission	2–3 weeks
Wanigasinghe et al. 2015 (11)	Single blind	97	1.4	Synthetic ACTH depot 40–60 IU every other day for 2 weeks (49)	Prednisolone 40–60 mg/day for 2 weeks (48)	Spasm cessation and EEG remission	Time taken to response; continued spasm control from day 14 to 42; quantitative reduction of spasms in non-responders	2 weeks
Gowda et al. 2019 (12)	Open label	34	1.6	ACTH 100 U/m^2^/day for 2 weeks (18)	Prednisolone 4 mg/kg/day (max 60 mg/day) for 2 weeks (16)	Spasm cessation and time taken to achieve spams cessation	Relapses and time taken to relapses; AEs; subsequent epilepsy rates	2 weeks
**ACTH DOSAGE**								
Hrachovy et al. 1994 (6)	Single blind	50	NR	ACTH 150 IU/m^2^/day for 3 weeks, 80 IU/m^2^/day for 2 weeks, 80 IU/m^2^ every other day for 3 weeks, 50 IU/m^2^ every other day for 1 week	ACTH 20–30 IU/day for 2 weeks	Spasm cessation and EEG remission	Relapses; AEs	2–6 weeks
Yanagaki et al. 1999 (8)	Open label	25	1.5	Synthetic ACTH 1 IU/kg/day for 2 weeks	Synthetic ACTH 0.2 IU/kg/day for 2 weeks	Spasm cessation and EEG remission	Relapses; AEs	2 weeks
Shu et al. 2009 (10)	Open label	30	1.3	ACTH 50 IU/day for 2 weeks	ACTH 0.4 IU/kg/day for 2 weeks	Spasm cessation and EEG remission	Relapses; AEs	2–6 weeks
**CORTICOSTEROID DOSAGE**								
Chellamuthu et al. 2014 (16)	Open label	63	2.3	Oral prednisolone in high dosage: 4 mg/kg/day for 2 weeks (31)	Oral prednisolone in usual dosage: 2 mg/kg/day for 2 weeks (32)	Spasm cessation	EEG remission; AEs	2 weeks
Kapoor et al. 2021 (17)	Open label	60	2.2	MEP 30 mg/kg/day for 3 days followed by oral prednisolone taper (31)	Oral prednisolone 4 mg/kg/day for 2 weeks (29)	Spasm cessation	Time taken to response; EEG remission at 2 and 6 weeks; AEs	2 weeks
**HORMONAL THERAPY VS. VGB**								
Vigevano et al. 1997 (18)	Open label	42	1.1	Depot ACTH 10 IU/day for 20 days (19)	VGB 100–150 mg/kg/day for 20 days (23)	Spasm cessation	EEG remission; time taken to response; AEs	2 weeks
Omar et al. 2002 (19)	Open label	32	1.3	ACTH 20 IU/day (16)	VGB average 87 mg/kg/day (16)	Spasm cessation	Time taken to response; AEs	NR
Lux et al. 2004 (9)	Open label	107	1.5	Tetracosactide depot 40–60 IU every other day for 2 weeks (25); prednisolone 40–60 mg/kg/day for 2 weeks (30)	VGB 100–150 mg/kg/day for 2 weeks (52)	Spasm cessation	Time taken to response; EEG remission	2 weeks
**HORMONAL THERAPY VS. OTHER TREATMENT**								
Dressler et al. 2019 (20)	PC-RCT; single blind	32	1.0	Synthetic ACTH 150 IU/m^2^ for 2 weeks (16)	Ketogenic diet (16)	Spasm cessation and EEG remission	Time taken to response; relapses; AEs; developmental outcomes	4 weeks
Angappan et al. 2019 (21)	Single blind	30	9	ACTH 30–60 IU/day for 2 weeks (15)	Zonisamide initial dosage: 4–8 mg/kg/day, max dosage: 25 mg/kg/day for 2 weeks (15)	Spasm cessation	EEG score; development quotient; AEs	At 2 and 6 weeks
**HORMONAL MONOTHERAPY VS. MULTI-THERAPY**								
Zou et al. 2010 (22)	Open label	38	1.5	ACTH 25 U/day for 3 weeks (19)	ACTH 25 U/day + MgSO_4_ 0.25 g/kg/day for 3 weeks (19)	Spasm cessation and EEG remission	Developmental outcome; AEs	At 4, 8, 12 and 24 weeks
O' Callaghan et al. 2017 (23)	Open label	377	1.3	Hormonal therapy alone: prednisolone 40–60 mg/day for 2 weeks or Tetracosactide depot 40–60 IU every other day for 2 weeks (191)	Hormonal therapy combined with VGB 100–150 mg/kg for 3 month (186)	Spasm cessation	Time taken to response; EEG remission; AEs	2–6 weeks
Kunnanayaka et al. 2018 (24)	Open label	62	2.4	Oral prednisolone 4 mg/kg/day (32)	Oral prednisolone combined with 30 mg/kg/day of pyridoxine (30)	Spasm cessation and EEG remission	AEs	2 weeks
Yi et al. 2019 (25)	Open label	77	2.2	Oral prednisone 40–60 mg/day for 2 weeks (39)	Oral prednisolone combined with TPM (moderate dosage 5 mg/kg/day for 5–6 weeks) (38)	Spasm cessation for 28 consecutive days	EEG remission at 2 weeks; development quotient; AEs	7–8 weeks

*NR, not reported; AE, adverse effect; PC-RCT, parallel-cohort randomized controlled trial*.

### Adrenocorticotrophic Hormone vs. Oral Corticosteroids

**Figure 3:**
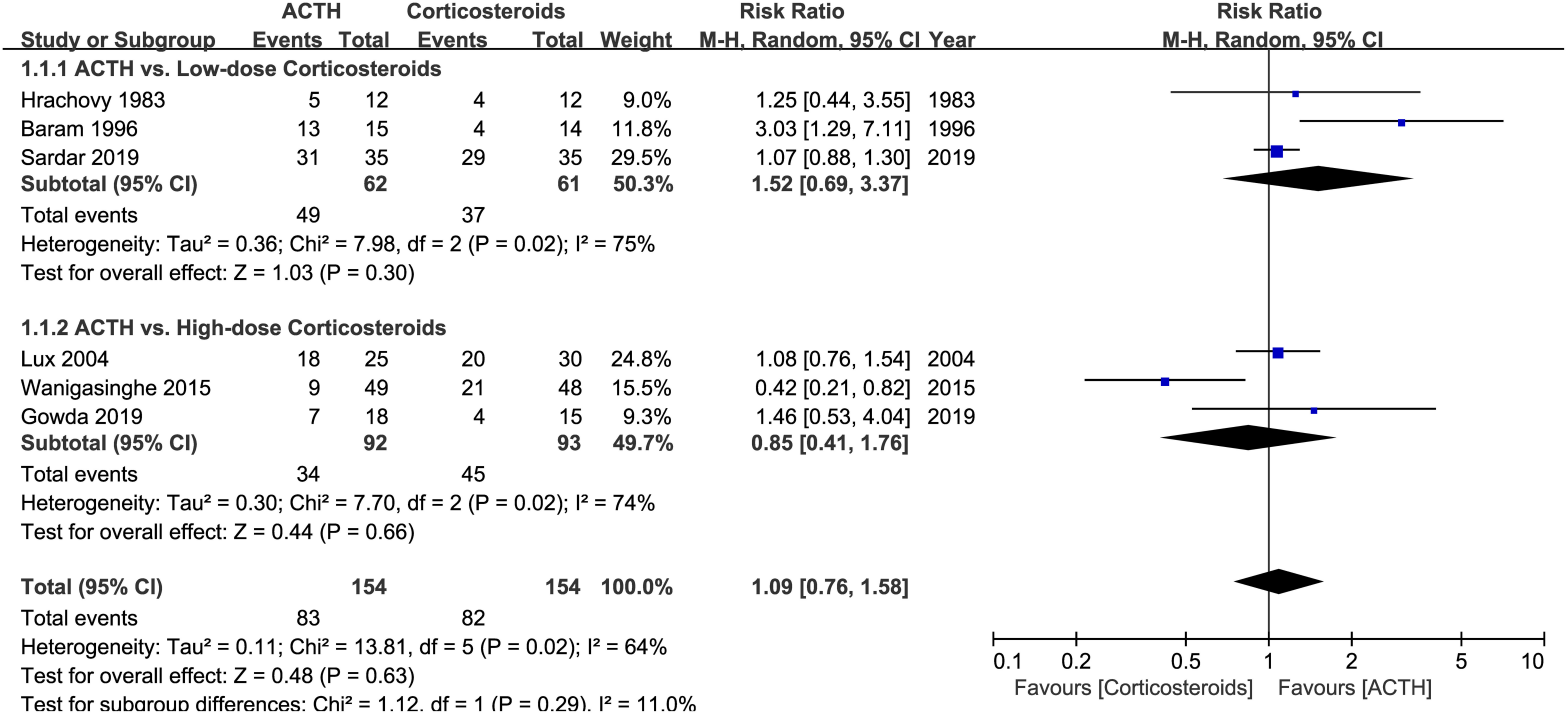
Forest plot showing risk ratio (RR) and 95% CI of electro-clinical response between adrenocorticotrophic hormone (ACTH) and oral corticosteroids.

Six RCTs that compared ACTH with oral corticosteroids for the treatment of ISs, with 323 participants included, met our inclusion criteria (5, 7, 9, 11, 12, 15). Three of these RCTs, namely, Hrachovy et al. (5), Baram et al. (7), and Sardar et al. (15), used low-dose corticosteroids (2 mg/kg/day of prednisone or prednisolone). The other three RCTs, namely, Lux et al. (9), Wanigasinghe et al. (11), and Gowda et al. (12), applied high-dose prednisolone (4 mg/kg/day or 40–60 mg/day). The forms (natural or synthetic analogs) and dosages of ACTH varied in these trials. The primary comparison of Lux et al. (9) was between hormonal therapy and VGB, so the secondary comparison of ACTH and prednisolone might be underpowered. Meta-analysis revealed that the effectiveness of ACTH and corticosteroids on electro-clinical response was comparable, regardless of the dosage of corticosteroids (Figure 3).

Hrachovy et al. (5) reported that six (25%) patients had hypertension (>140/90 mmHg) during the initial and crossover stages, of which four developed at both stages and two developed only when receiving prednisone. After 2 weeks of the initial stages, 10 of 16 patients who received serial CT scans showed evidence of cerebral shrinkage, such as increased ventricular size and/or increased subarachnoid space, but no comparisons were made between treatment groups. Lux et al. (9) reported that 11 (36.7%) patients in the prednisolone group and 8 (32.0%) in the ACTH group had hypertension (>120/90 mmHg). Glycosuria was found in one patient in the ACTH group. In Gowda et al. (12), the incidence of AEs in the prednisolone group was 20% compared to 16.6% in the ACTH group, but details about the profile of AEs were not reported. Wanigasinghe et al. (11) found a higher incidence of abdominal distension in the prednisolone group (21.1% vs. 0, adjusted *p*-value <0.00294), whereas the other AEs, such as increased appetite, weight gain, Cushingoid features, insomnolence, hypertension, increased susceptibility to infection, and electrolyte imbalances, were not different between two groups.

### Adrenocorticotrophic Hormone Dosage

**Figure 4:**
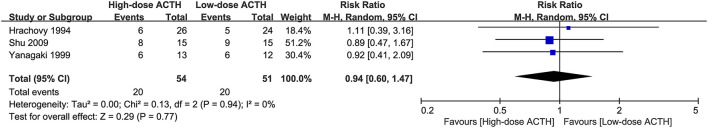
Forest plot showing RR and 95% CI of electro-clinical response between high and low dose of ACTH.

**Figure 5:**
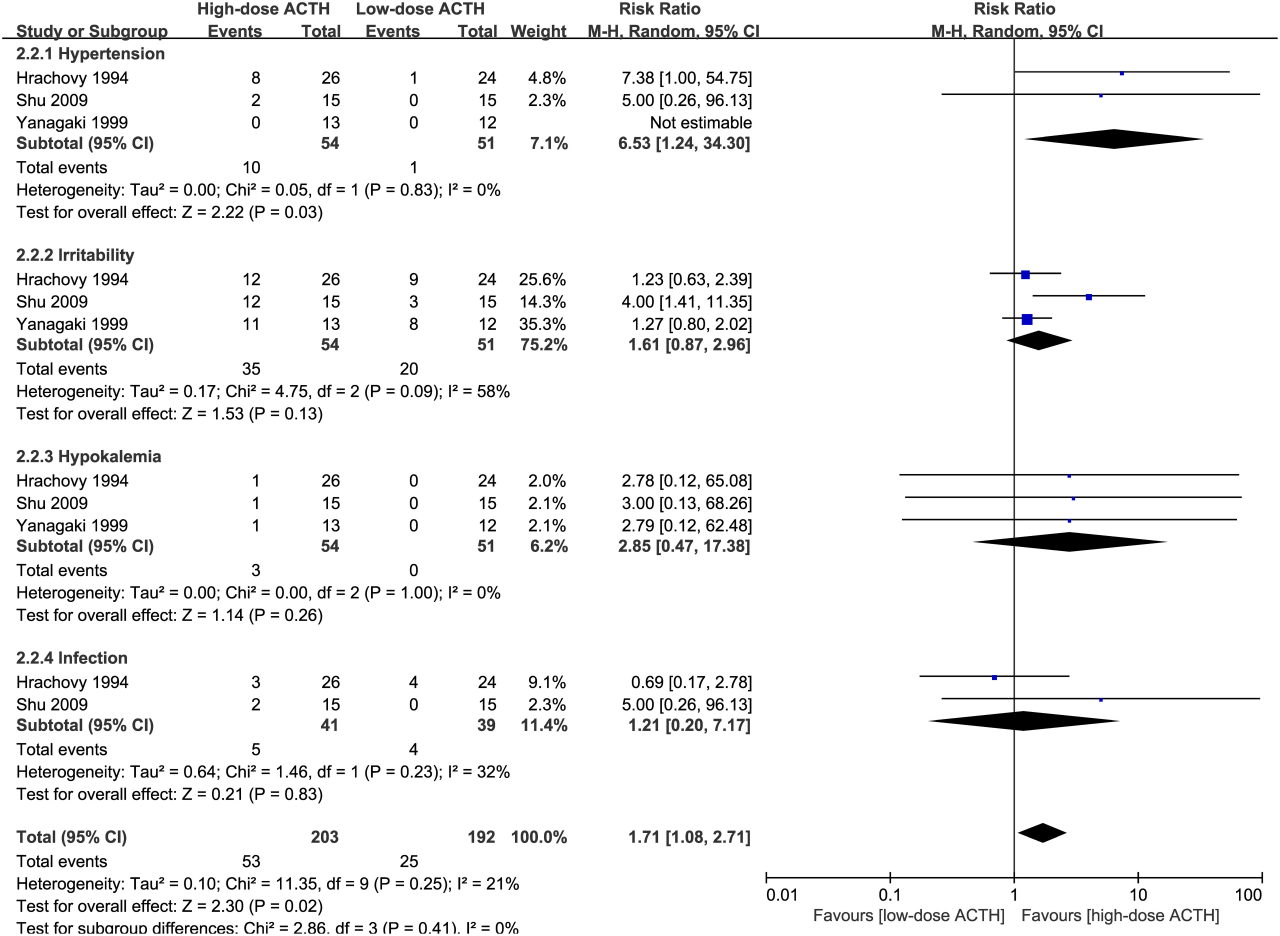
Forest plot showing RR and 95% CI of adverse effects between high and low dose of ACTH.

Three RCTs that compared the effectiveness and safety of different dosages of ACTH were included in our analysis (6, 8, 10). There was no significant difference in the proportion of patients achieving electro-clinical response between high- and low-dose groups (RR = 0.94, 95% CI 0.60–1.47, Figure 4). However, the patients in the high-dose ACTH treatment group had a higher incidence rate of AEs (RR = 1.71, 95% CI 1.08–2.71, Figure 5). Except for the AEs shown in Figure 5, Yanagaki et al. (8) and Shu et al. (10) also reported brain shrinkage but in different measurement methods. Shu et al. (10) reported one case of brain shrinkage in the high-dose ACTH group but no case in the low-dose group, whereas Yanagaki et al. (8) evaluated the volume difference of lateral ventricles, showing brain shrinkage was significantly milder in the low-dose group than in the high-dose group (6.6 ± 4.6 vs. 12.4 ± 5.7%; *p* < 0.05).

### Corticosteroid Dosage

An open-label RCT, Chellamuthu et al. (16), compared the effectiveness and safety of high- (4 mg/kg/day) and usual-dose (2 mg/kg/day) prednisolone for the treatment of ISs. A total of 63 participants, aged 3–24 months, were enrolled, with 31 randomly allocated to the high-dose group and 32 to the usual-dose group. On day 14 after the initiation of treatment, 12 (38.7%) and 7 (21.9%) of participants achieved electro-clinical response in high- and usual-dose groups, respectively (*p* = 0.15). The occurrence of AEs, including increased appetite, weight gain, Cushingoid features, and infections, was higher in the high-dose group but did not reach a statistical significance.

A recent open-label RCT, Kapoor et al. (17) explored the feasibility of shortening the treatment course by using methylprednisolone pulses (MEP). By day 14, no difference was found in the proportion of patients achieving spasm cessation (17/31 vs. 20/29, *p* = 0.26) and electro-clinical response between MEP and oral prednisolone groups; however, no details about EEG remission were given. Patients in the MEP group took less time to achieve remission (mean 5.4 ± 0.9 vs. 9.5 ± 2.6 days, *p* < 0.0001). However, 6 (19.4%) patients who received MEP had spasm recurrence, compared to none in the oral prednisolone group. Therefore, by 6 weeks, the proportion of patients who had EEG remission was significantly lower in the MEP group (45.2 vs. 75.9%, *p* < 0.015). Increased appetite (65.5 vs. 3.2%) and weight gain (75.9 vs. 16.1%) were more common in oral prednisolone group (*p* < 0.05), whereas sleep disturbance (61.3 vs. 20.7%, *p* = 0.0014), hypertension (35.5 and 10.3%, *p* = 0.032), and irritability (74.2 vs. 31%, *p* = 0.0008) were more frequent in MEP group.

### Hormonal Therapy vs. VGB

**Figure 6:**
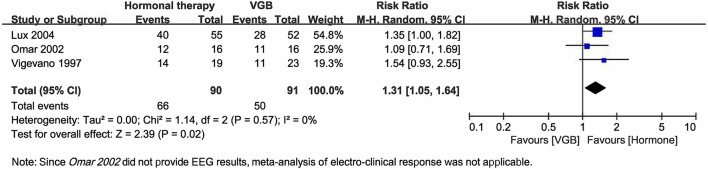
Forest plot showing RR and 95% CI of spasm cessation between hormonal therapy and vigabatrin.

Of three included RCTs that compared hormonal therapy to VGB (9, 19, 26), Vigevano and Cilio (26) included 4 patients with tuberous sclerosis complex (TSC), with three assigned to VGB arm and one assigned to hormone therapy arm and all four patients responded to treatments, whereas Omar et al. (19) included 2 patients with TSC without reporting treatment assignment and response. The combined result showed that ACTH had a more favorable effect than VGB on cessation of spasms (RR = 1.31, 95% CI 1.05–1.64, Figure 6). Both Vigevano and Cilio (26) and Lux et al. (9) summarized the short-term AEs, demonstrating that the number of overall AEs was similar to hormonal therapy (*n* = 62) and VGB (*n* = 55). Patients showed hypertension, increased appetite, irritability on hormonal therapy, and more drowsiness on VGB. No visual effects were detected during 2 weeks of monitoring. Omar et al. (19) did a long-term follow-up with visual evoked responses and electroretinography carried out by an ophthalmologist and found no significant changes.

### Adrenocorticotrophic Hormone vs. Other Treatments

Dressler et al. (20) was a parallel-cohort (PC) RCT comparing KD and high-dose synthetic ACTH for ISs with 32 patients assigned in the RCT (16 KD, 16 ACTH). The other 69 patients who were not eligible for the RCT were allocated in the PC (37 KD and 32 ACTH). The proportion of patients achieving the primary outcome, i.e., electro-clinical remission, was similar between KD and ACTH groups (RCT 62 vs. 69%; PC 41 vs. 38%; combined cohort 47 vs. 48%). There was also no difference in time taken to electro-clinical remission between KD and ACTH groups. The relapse rate was similar between treatment arms in the RCT but lower for KD in the PC (KD 0 vs. ACTH 50%, *p* < 0.001). The frequency of overall AEs was not different between KD and ACTH groups (42 vs. 45%), but medical interventions to treat AEs were significantly less required in the KD group (30 vs. 94%, *p* < 0.001).

Another RCT investigated the effectiveness of oral zonisamide for ISs compared to ACTH (21). At 14 days of the initiation of treatment, the proportions of children with electro-clinical responses were not statistically different in the two groups (zonisamide 3/15 vs. ACTH 5/15, *p* = 0.68). At 6 weeks of treatment, more patients had EEG remission (zonisamide 5/14 vs. ACTH 10/14, *p* = 0.14). Patients of the ACTH group had significantly better hypsarrhythmia scores, which were assessed *via* the Jeavons scoring system. Predominant AEs of patients receiving zonisamide were lethargy and irritability. One case required dose reduction due to metabolic acidosis, and one case withdrew the medication due to significant loss of appetite.

### Monotherapy vs. Multitherapy

Zou et al. (22) was an open-label RCT with a 24-week follow-up duration, comparing the effectiveness of ACTH monotherapy and ACTH with MgSO_4_. At 4 weeks of the initiation of treatment, the proportion of patients with spasm cessation was higher in ACTH + MgSO_4_ group compared to the ACTH group (12/19 vs. 8/19, *p* = 0.003), but the proportions of patients with electro-clinical response were not statistically different in the two groups. Developmental quotients (DQs) measured by the Gesell tests showed no difference between the two groups at baseline and after 24 weeks of treatment. However, patients of the combination therapy group showed a significant improvement of mean DQ at personal-social dimension compared to baseline level (combination therapy group: from 48.6 to 65.2, *p* < 0.05; ACTH group: from 47.7 to 49.9). AEs were reported in 31.6% and 42.1% of patients in the combination therapy group and ACTH group, respectively, but were not significantly different between the two groups.

O'Callaghan et al. (23) was an open-label RCT that studied whether the combination of hormonal therapy and VGB was superior to hormonal therapy alone. The type of hormonal therapy (i.e., prednisolone or tetracosactide depot) was randomly assigned or chosen by parents. Spasm cessation on days 13 and 14 was achieved in 166 (89%) of 186 patients treated with combination therapy compared to 132 (69%) of 191 patients on hormonal therapy (95% CI 11.8–28.6; χ^2^ = 23.2; *p* < 0.001). Electro-clinical remission was achieved in 123 (66%) patients on combination therapy compared to 104 (55%) who were allocated to hormonal therapy (95% CI 1.4–21.6; χ^2^ = 5.2; *p* = 0.023). Time taken to reach spasm-free was shorter on combination therapy than hormonal therapy [median 2 days (interquartile range (IQR) 2–4) vs. median 4 days (IQR 3–6); *z* = 6.04; *p* < 0.001]. No significant difference in AEs was found between groups (117 in the combination therapy group and 111 in the hormonal therapy group).

Kunnanayaka et al. (24) was an open-label RCT to explore whether the addition of pyridoxine to prednisolone could offer more benefits to patients with ISs. All 66 eligible patients received 30 mg/kg/day of pyridoxine for 3 days before the commencement of the study. Four patients were excluded due to marked improvement after the initial pyridoxine trial. The proportions of children with electro-clinical remission on day 14 were not significantly different (monotherapy 28.1% vs. combination therapy 30.0%, *p* = 0.87). The AEs in both groups included increased appetite, irritability, weight gain, Cushingoid facies, and excessive daytime sleepiness, but no patients were required to withdraw the treatments. Follow-up of the patients who were spasm-free showed that 4 patients relapsed in the prednisolone group and one relapsed in the combination therapy group after 1 month of treatment.

Yi et al. (25) was an RCT that studied whether the addition of topiramate (TPM) to prednisone was more beneficial for patients with ISs or late-onset epileptic spasms. Cessation of spams at day 14 after treatment entry was achieved in 28 (71.8%) of 39 patients on prednisone and in 29 (76.3%) of 38 patients on combination therapy (χ^2^ = 0.205; *p* = 0.796). Among these patients who were spasm-free, 21 of 28 patients in the monotherapy group and 20 of 29 in the combination therapy group achieved electro-clinical remission. The rate of patients who stayed spasm-free in the monotherapy group and the combination therapy group at day 49 or 56 was not significantly different (71.8 vs. 65.8%, *p* = 0.569), so was at day 120 (61.5 vs. 50.0%, *p* = 0.308). There was no significant difference in the rate of patients occurring AEs between the two treatment groups.

### Network Meta-Analysis

**Figure 7:**
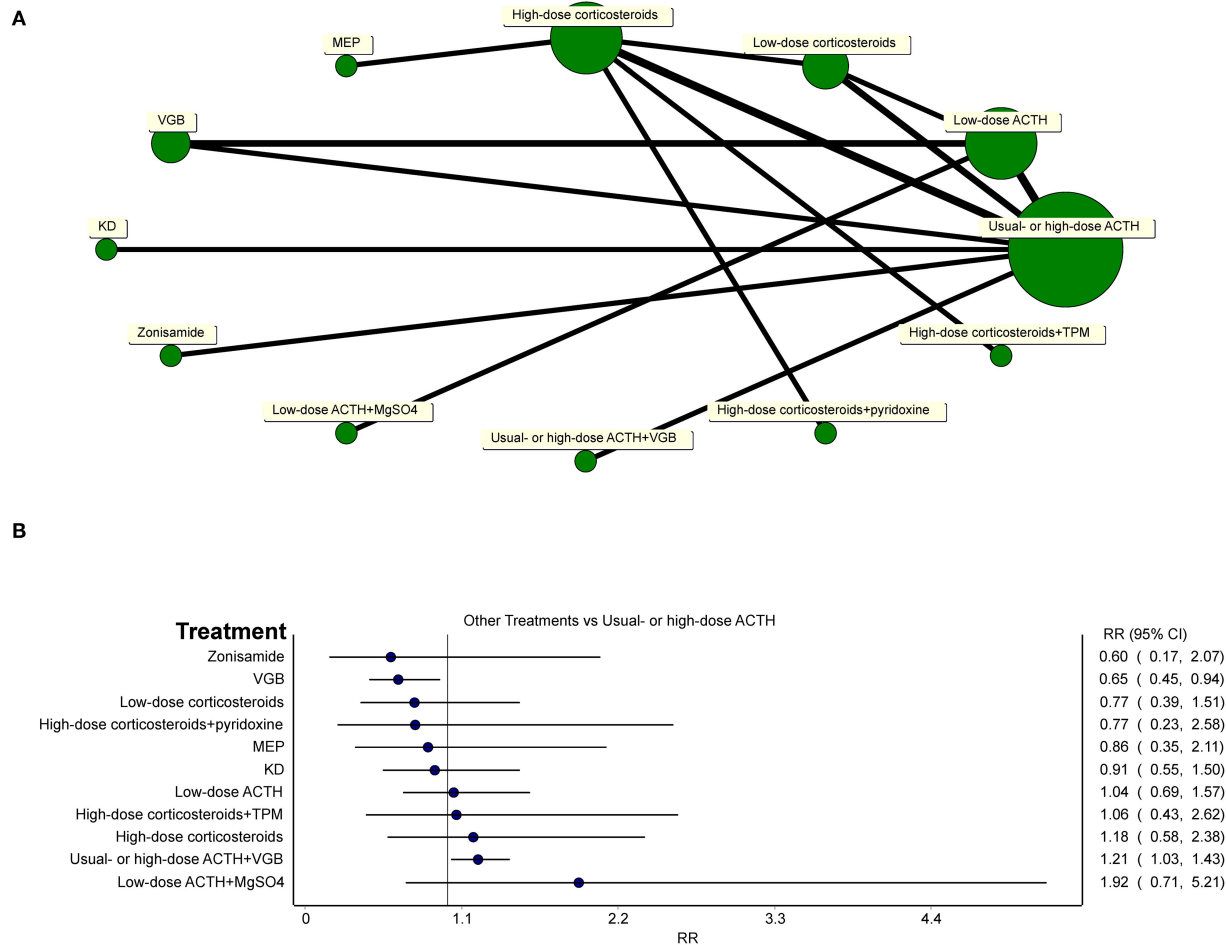
**(A)** Network of all treatments of included randomized controlled trials. **(B)** Network meta-analysis of all other treatments vs. usual-dose ACTH in the effectiveness of the electro-clinical response.

To gain a comprehensive understanding of the efficacy of all included treatments compared to usual- or high-dose ACTH, a network meta-analysis was conducted using MetaXL (version 5.3) (Figure 7A). Because ACTH usage was rather heterogeneous, we defined less than 25 U/day of ACTH as low-dose ACTH, otherwise as usual- or high-dose ACTH. The assignment of treatment groups of all studies and all comparisons of network meta-analysis are provided in Supplementary Material 2.

Network meta-analysis (Figure 7B) showed that usual- or high-dose ACTH did not superior to oral corticosteroids (regardless of the dosage of oral corticosteroids), MEP, zonisamide, and KD in the effectiveness of the electro-clinical response. Additional treatments to hormonal therapy, such as TPM, pyridoxine, and MgSO_4_, did not lend any benefits to patients in controlling spasms, but the combination of hormonal therapy and VGB was better than using ACTH alone (RR = 1.21, 95% CI 1.03–1.43). For general patients without TSC, ACTH had an edge over VGB (RR = 0.65, 95% CI 0.45–0.94).

## Discussion

The limitations of ACTH treatment for ISs, such as high costs, availability in resource-limited countries, and severe AEs, make good alternatives of oral corticosteroids. Especially, there is currently no potent evidence showing ACTH is superior to oral corticosteroids regarding spasm cessation and EEG remission of hypsarrhythmia. Our meta-analysis showed that oral corticosteroids, prednisone, and prednisolone, regardless of low (2 mg/kg/day) or high dosage (40–60 mg/day or 4 mg/kg/day), were as effective as ACTH in controlling spasms. However, considering that infants have the inadequate ability of the HSD11B1 enzyme to reduce prednisone to prednisolone, the use of low-dose prednisone probably should not be recommended (27). Although oral corticosteroids have overcome many of the limitations associated with ACTH treatment, their overall long-period treatment (usually 4–6 weeks) is still a drawback. MEP, as another application form of corticosteroids, overcomes the drawback of long-period treatment and, thus, improves treatment compliance. No head-to-head RCT that compared ACTH and MEP was retrieved during literature searching, but through network meta-analysis, we found that MEP had similar efficacy as ACTH in the short-term spasm control (RR = 0.86, 95% CI 0.35–2.11). However, the included RCT (17) and a non-RCT (28) both reported a higher rate of reoccurrence of spasms in patients who received MEP, which should not be overlooked.

The commonly reported serious AEs of ACTH treatment include hypertension, infection, reversible brain shrinkage, cardiac hypertrophy, and electrolyte abnormalities, which deter the extensive usage of ACTH. These AEs may be dose- and duration-dependent, so it is of great concern to strike the balance among the lower ACTH dose, fewer AEs, and maximum therapeutic effects. Our analysis showed the effectiveness of low- and usual-dose ACTH was not different, but patients using usual-dose ACTH were at higher risk of having AEs. Overall, low-dose ACTH should be considered over high-dose regimens due to comparative effectiveness and a preferable profile of AEs.

The VGB, as an inhibitor of GABA-transaminase, has been introduced as a first-choice drug in the treatment of ISs, especially it is the best choice for IS caused by TSC (29, 30). VGB is given orally and rapidly absorbed, which is a great advantage for pediatric patients. Although visual-field defect, which is usually irreversible and asymptomatic, has been reported in pediatric patients, VGB, in general, is well-tolerated in children (31). It is of utmost importance to address whether VGB has similar therapeutic effectiveness as hormonal therapy in patients with IS who do not have TSC. Our analysis showed that hormonal therapy had better efficacy in spasm cessation for general patients with ISs; compared to VGB, even a few patients with TSC were occasionally included. In addition, the only regimen in our analysis that had better efficacy than ACTH was the combined use of hormonal therapy and VGB.

Even if hormonal therapy and VGB are administered at the first-line treatments, only about 30% of patients achieve long-term remission. Many patients have the reoccurrence of spasms or develop other types of epilepsy, such as Lennox-Gastaut Syndrome. There is a strong demand for other compelling therapeutic options. A systematic review showed that at least 25% of patients, most of which failed the first-line treatments, can be expected to have a >50% reduction of spasm frequency after the treatment of zonisamide (32). A systematic review of 13 observational studies showed that 57.4% of patients achieved >50% improvement in spasm frequency with 6 months of KD treatment (33). A recent non-RCT on patients who failed hormonal therapy showed that 13.4% of patients had ≥50% spasm reduction compared to 10% in the control group (34). When addressing the effectiveness and safety of other non-first-line treatment for ISs, out of consideration of ethics, most studies used observational or uncontrolled trials, and inevitably, the included patients often have complicated medication histories of AEDs or long treatment lag. During our literature search, only two RCTs administered alternative treatments (i.e., KD and zonisamide) as the first-line treatments to patients with hormonal therapy naïve. Our network meta-analysis revealed that neither of these two treatments showed a difference in the effectiveness of short-term spasm control compared to usual-dose ACTH. Additionally, the combining use of hormonal therapy with TPM, pyridoxine, or MgSO_4_ did not lend any benefits to patients in spasm control, either.

The main limitation of this study is the high heterogeneity of included patients and treatment protocols. The age range, sex ratio, and the ratio of cryptogenic and symptomatic ISs varied from study to study. A variety of agents were used, such as natural ACTH gel or synthetic ACTH, in a rather wide range of treatment lengths and dosages. Although 2 weeks after initiation of treatment was the most common time point to evaluate electro-clinical response, some studies adopted longer time intervals (4–8 weeks) before the evaluation, which was another source of clinical heterogeneity. As shown in Zou et al. (22) and Angappan et al. (21), the proportion of patients with EEG remission increased at a later time point. Another limitation is the limited number and small sample size of RCTs that studied other alternative treatments, such as KD or zonisamide, thereby, making the level of evidence low. Furthermore, many of the included studies, even studies that compared hormonal therapy and VGB, did not exclude the patients with TSC, which may confound the comparison of effectiveness. Thus, our results must be interpreted with caution.

## Conclusion

Our systematic review and meta-analysis showed that oral corticosteroids could be optional alternatives when ACTH is not applicable due to high costs, limited availability, or severe AEs, but for MEP as the first-line treatment, more studies are needed to explore the treatment duration and dosage and continuous effects on spasm control. Low-dose ACTH is recommended because of similar effectiveness and lower risk of AEs compared to high-dose ACTH. The evaluation of the effectiveness of other alternative treatments still needs RCTs with multicentric involvement and larger sample size to provide a higher level of evidence.

## Data Availability Statement

The original contributions presented in the study are included in the article/Supplementary Material, further inquiries can be directed to the corresponding author/s.

## Author Contributions

SG developed the literature search protocol, conducted data analysis, and drafted and revised the manuscript for intellectual content. SG and LM conducted a literature search. SG and LZ contributed to data extraction. FL and ZP contributed to the assessment of risks of bias. FY revised the manuscript for intellectual content. JP planned, designed, and conceptualized this study and revised the manuscript for intellectual content. All authors contributed to the article and approved the submitted version.

## Funding

This study was supported by grants from the National Natural Science Foundation of China (Grant Nos. 81771409 and 82071462).

## Conflict of Interest

The authors declare that the research was conducted in the absence of any commercial or financial relationships that could be construed as a potential conflict of interest.

## Publisher's Note

All claims expressed in this article are solely those of the authors and do not necessarily represent those of their affiliated organizations, or those of the publisher, the editors and the reviewers. Any product that may be evaluated in this article, or claim that may be made by its manufacturer, is not guaranteed or endorsed by the publisher.
