# Supplementary material for: Hormonal Therapy for Infantile Spasms: A Systematic Review and Meta-Analysis
Source: Front Neurol. 2022 Feb 10;13:772333. doi: 10.3389/fneur.2022.772333 (PMC8867209; doi:10.3389/fneur.2022.772333)
Supplement: Supplementary file 1 [file Table_1.DOCX]

Embase (377 results)

#1: 'infantile spasm'/exp OR 'infantile spasm'

#2: 'infantile spasms':ti,ab,kw OR 'west syndrome':ti,ab,kw OR 'infantile myoclonic':ti,ab,kw OR 'flexor spasm':ti,ab,kw OR 'infantile myoclonic encephalopathy':ti,ab,kw OR 'myoclonic infantile encephalopathy':ti,ab,kw OR 'infantile spasmus':ti,ab,kw OR 'spasm, infantile':ti,ab,kw OR 'spasms, infantile':ti,ab,kw OR 'propulsive petit mal':ti,ab,kw OR 'jack knife seizure':ti,ab,kw

#3: 'corticosteroid'/exp OR 'corticosteroid' OR 'corticotropin'/exp OR 'corticotropin'

#4: corticosteroids:ti,ab,kw OR 'adrenal cortex hormone*':ti,ab,kw OR 'adrenal cortical hormone*':ti,ab,kw OR 'adrenal cortical steroid':ti,ab,kw OR 'adrenal steroid':ti,ab,kw OR 'adrenal steroid hormone':ti,ab,kw OR 'adreno cortical steroid':ti,ab,kw OR 'adrenocortical hormone':ti,ab,kw OR 'adreno corticosteroid':ti,ab,kw OR 'adrenocortical steroid':ti,ab,kw OR adrenocorticosteroid:ti,ab,kw OR acth:ti,ab,kw OR 'adrenal cortex trop*ic hormone':ti,ab,kw OR 'adrenocorticotrop*ic hormone':ti,ab,kw OR adrenocorticotrophin:ti,ab,kw

#5: 'controlled study'/exp OR 'controlled study'

#6: 'controlled trial':ti,ab,kw OR 'controlled clinical trial':ti,ab,kw OR 'controlled clinical study':ti,ab,kw

#7: #1 OR #2

#8: #3 OR #4

#9: #5 OR #6

#10: #7 AND #8 AND #9

Ovid MEDLINE (R) 1946 – present (42 resutls)

#1: infantile spasm.mp. or exp Spasms, Infantile/

#2: (infantile spasm* or west syndrome or lightning attack* or hypsarrhythmia* or jack knife seizure* or nodding spasm*).ab.

#3: corticosteroids.mp. or exp Adrenal Cortex Hormones/

#4: (corticosteroid* or corticoid* or adrenal cortical hormone or adrenal cortical steroid or adrenal steroid or adrenocortical hormone or adreno corticosteroid).ab.

#5: acth.mp. or exp Adrenocorticotropic Hormone/

#6: (Corticotrop*in or adrenocorticotrop*ic Hormone or acth).ab.

#7: Controlled Clinical Trial.mp. or exp Controlled Clinical Trial/

#8: (controlled trial or controlled study or controlled clinical study).ab.

#9: #1 OR #2

#10: #3 OR #4 OR #5 OR #6

#11: #7 OR #8

#12: #9 AND #10 AND #11

CENTRAL (87 results)

#1: MeSH descriptor: [Adrenal Cortex Hormones] explode all trees

#2: MeSH descriptor: [Steroids] explode all trees

#3: MeSH descriptor: [Adrenocorticotropic Hormone] explode all trees

#4: (hormones):ti,ab,kw OR ("corticosteroid"):ti,ab,kw OR (corticoid):ti,ab,kw OR ("glucocorticoid"):ti,ab,kw

#5: (prednisone):ti,ab,kw OR ("prednisolone"):ti,ab,kw OR ("methylprednisolone"):ti,ab,kw OR (ACTH):ti,ab,kw

#6: ("corticotropin"):ti,ab,kw OR ("corticotrophin"):ti,ab,kw OR ("adrenocorticotrophin"):ti,ab,kw OR ("adrenocorticotropin"):ti,ab,kw OR ("tetracosactide"):ti,ab,kw

#7: ("tetracosactrin"):ti,ab,kw OR ("tetracosapeptide"):ti,ab,kw OR ("Cortrosyn"):ti,ab,kw OR ("Cortosyn"):ti,ab,kw

#8: #1 OR #2 OR #3 OR #4 OR #5 OR #6 OR #7

#9: MeSH descriptor: [Spasms, Infantile] explode all trees

#10: (encephalopathy, infantile myoclonic):ti,ab,kw OR (myoclonic infantile encephalopathy):ti,ab,kw OR (infantile myoclonic encephalopathy):ti,ab,kw OR ("infantile spasm"):ti,ab,kw OR (infantile spasmus):ti,ab,kw

#11: ("jack knife seizure"):ti,ab,kw OR (minor motor epilepsy):ti,ab,kw OR (spasm in flexion):ti,ab,kw OR (flexor spasm):ti,ab,kw AND ("West syndrome"):ti,ab,kw

#12: #9 OR #10 OR #11

#13: #8 AND #12
